# Prevalence of UL97 gene mutations and polymorphisms in cytomegalovirus infection in the colon associated with or without ulcerative colitis

**DOI:** 10.1038/s41598-021-93168-x

**Published:** 2021-07-01

**Authors:** Satoshi Tamura, Satoshi Osawa, Natsuki Ishida, Takahiro Miyazu, Shinya Tani, Mihoko Yamade, Moriya Iwaizumi, Yasushi Hamaya, Isao Kosugi, Takahisa Furuta, Ken Sugimoto

**Affiliations:** 1 Department of Endoscopic and Photodynamic Medicine, Hamamatsu University School of Medicine, 1-20-1 Handayama, Higashi-ku, Hamamatsu, 431-3192 Japan; 2 First Department of Medicine, Hamamatsu University School of Medicine, 1-20-1 Handayama, Higashi-ku, Hamamatsu, 431-3192 Japan; 3 Department of Laboratory Medicine, Hamamatsu University School of Medicine, 1-20-1 Handayama, Higashi-ku, Hamamatsu, 431-3192 Japan; 4 Department of Regenerative and Infectious Pathology, Hamamatsu University School of Medicine, 1-20-1 Handayama, Higashi-ku, Hamamatsu, 431-3192 Japan; 5 Center for Clinical Research, Hamamatsu University School of Medicine, 1-20-1 Handayama, Higashi-ku, Hamamatsu, 431-3192 Japan

**Keywords:** Translational research, Epidemiology, Inflammatory bowel disease, Ulcerative colitis

## Abstract

Cytomegalovirus (CMV) reactivation in the colon is common in patients with severe ulcerative colitis (UC). Ganciclovir (GCV) resistance conferring CMV UL97 gene mutations have been reported in recent years. However, the prevalence of UL97 gene mutations in GCV-naive CMV infection in the colon remains unknown. We investigated the prevalence of CMV UL97 gene mutations in patients with colonic CMV infection associated with or without UC. Twenty-two GCV-naive patients with colonic CMV infection, 15 with UC and 7 with other diseases, were enrolled. Frozen biopsy samples or formalin-fixed paraffin-embedded samples were used for nested polymerase chain reaction (PCR) amplification of the UL97 gene. Sanger DNA sequencing was performed. In comparison with AD169 reference strain, natural polymorphisms were frequently detected in codons N68D (100%), I244V (100%), and D605E (86.4%). Seven polymorphisms were detected infrequently (< 10%) outside the kinase domain. However, no known GCV resistance mutations were found. There seemed to be no difference between the ratio of polymorphisms in patients with and without UC. In conclusions, we did not detect UL97 gene mutations associated with GCV resistance in GCV-naive patients with or without UC. Consistent with previous reports, D605E polymorphism may be used as a genetic marker for CMV in East Asian countries.

## Introduction

Human cytomegalovirus (CMV) infections cause significant morbidity and mortality in immunocompromised hosts, such as patients who have undergone solid organ or bone marrow transplantation, human immunodeficiency virus (HIV)-infected patients, and children with congenital immunodeficiencies^1,2^. In patients with flare­ups of refractory ulcerative colitis (UC) and sometimes in immunocompromised patients, CMV reactivation in the colon is common and may be associated with poor prognosis^3^. In immunocompetent individuals, CMV usually causes self-limiting mild hepatitis, mononucleosis, or subclinical infection^4^. Antiviral treatment with ganciclovir (GCV) has been highly recommended for CMV reactivation, along with anti-tumor necrosis factor (anti-TNF) monoclonal antibody therapy, without losing time, even though there is some debate recently in flare­ups of refractory UC^5–9^.

GCV, a 2′-deoxyguanosine nucleoside analog, was used as the first-line drug for the treatment of CMV disease and for prophylaxis in groups at high risk for CMV infection. However, prolonged therapy with GCV can lead to the development of GCV-resistant mutations^10,11^. GCV is selectively phosphorylated by a viral protein kinase homolog, a product of the UL97 gene^12,13^. Approximately 90% of GCV resistance results from mutations in that gene^13^. Well characterized GCV resistance mutations at UL97 codons 460, 520, and 590–607 impair the phosphorylation of GCV that is necessary for its antiviral activity, presumably by altering substrate recognition^14–16^. Despite their lack of association with GCV resistance, polymorphism likely to be related to regionality have also been reported^17,18^.

Previous studies have revealed the frequency of drug-resistant CMV in organ transplant patients, bone marrow transplant patients, and HIV-infected patients, and have discussed algorithms for antiviral therapy^11,19^. However, there are no data regarding the prevalence of UL97 gene mutations in colonic CMV infection associated with or without UC. One of the clinical questions is whether or not the uniform use of GCV is appropriate for initial antiviral therapy. The aim of this study was to investigate the prevalence of GCV resistance-conferring UL97 gene mutations in Japanese patients with colonic CMV infection associated with or without UC, especially in GCV-naive patients.

## Methods

### Study design

This was a single-center, retrospective study conducted in patients with colonic CMV infections with and without UC. This study was conducted ethically based on the Declaration of Helsinki. In accordance with the Ethical Guidelines for Medical and Health Research Involving Human Subjects (Ministry of Education, Culture, Sports, Science and Technology and Ministry of Health, Labour and Welfare, Japan), informed consent was omitted, and information of this study was disclosed in the form of an opt-out on our hospital website. Information regarding the conduct of the research, including the objectives, was disclosed, and the research subjects were provided an opportunity to refuse inclusion in the research. The study protocol involving these issues was reviewed and approved by the Ethics Committee of Hamamatsu University School of Medicine, Japan (EG16-257).

### Patients and specimens

Twenty-two GCV-naive patients who were diagnosed with colonic CMV infection in our hospital between 2012 and 2018 were enrolled in this study. Colonic CMV infection was defined as virus isolation or detection of viral proteins (antigens) or nucleic acid in colonic tissue specimen^20^. Immunohistochemical staining of CMV was performed with mouse monoclonal antibody (code M0854, clones DDG9/CCH2; Dako, Glostrup, Denmark). Fifteen patients with CMV infection in the colon had UC, and seven patients had other diseases. Frozen biopsy samples or formalin-fixed paraffin-embedded samples were used for nested polymerase chain reaction (PCR) amplification of the UL97 gene.

### DNA purification from the samples

Frozen biopsy samples were used for DNA extraction using the DNeasy Blood and Tissue Kit (Qiagen, Germantown, MD). Formalin-fixed paraffin-embedded samples were purified genomic DNA using the QIAamp DNA FFPE Tissue Kit (Qiagen). The extractions were carried out according to the manufacturer’s instructions.

### Nested PCR amplification

**Figure 1:**
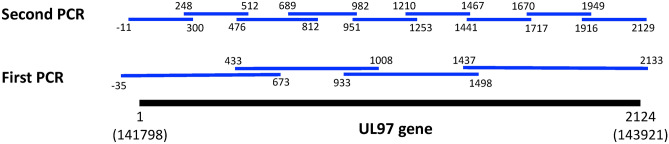
Diagram of nested PCR primer sites for UL97 gene analysis to determine GCV resistance mutation. The full length of UL97 gene (2123 bp) is presented at the bottom. The initial nested PCR primer sites are represented in the lower row. The second PCR primer sites for DNA sequencing are represented in the upper row.

All PCR primers were purchased from Merck KGaA (Darmstadt, Germany). Nested PCR was performed with KOD FX Neo (TOYOBO CO., LTD, Osaka, Japan) to reduce polymerase errors. The primer sets used in the nested PCR for this study are shown in Fig. 1 and Table 1. The UL97 fragment was amplified using 0.4 mM dNTP, 1 U KOD FX Neo, 2X PCR buffer, 300 nM of each primer, and 50 ng genomic DNA of CMV. For the first round of PCR, the thermal cycler was run at 98 °C for 10 s, 58 °C for 30 s and 72 °C for 30 s for 25 cycles, then 72 °C for 7 min. For the second round of PCR, the thermal cycler was run at 98 °C for 10 s, 62 °C for 30 s, and 72 °C for 30 s for 25 cycles, then 72 °C for 7 min. The QIAquick PCR Purification Kit (Qiagen) was used to purify the PCR products. Reaction products were stored at − 20 °C until they were analyzed by 2% agarose gel electrophoresis or purified for DNA sequencing.

**Table 1:** Primer sets used in the nested PCR for this study.

Primer name	Sequence (5′–3′)		Nucleotides	Product length (bp)
	Forward	Reverse		
**1st round PCR**				
UL97 N1	GTGCAGCCCTAGGAACA	CGTTTTCTTCGAGCACC	− 35 to 673	708
UL97 N2	GGTTACCACGGCTTGC	GCGGTAGCTCTCGTCG	433–1008	575
UL97 N3	GGTCTACGTGCCCAAAGA	GAAAGACGGCCACACAG	933–1498	565
UL97 N3	GTGCGATTACAGCCTCAG	GCGTCCAGGTTACTCG	1437–2133	696
**2nd round PCR**				
UL97_1	CTGTCGCCACTATGTCCTCC	GAACGCATGCGGAAAAAGTC	− 11 to 300	311
UL97_2	CCACTTTGACCACCCTGAGT	GTGAAGAGAGCGCGGCGTA	248–512	264
UL97_3	CGTTCGAGTACGATCGCGAC	GTCGTTGGAACAGGTGCAAT	476–812	336
UL97_4	CGGAAAGTCAGGACAGCG	CTCGTCGCTCATGTCCAC	689–982	293
UL97_5	GGACGATTTTTGCCACAAGA	CCACTGGTCGTGATGAAACA	951–1253	302
UL97_6	CTGCTGCACAACGTCACGGT	CACAGCGCTCGTTGTAATCC	1210–1467	257
UL97_7	GATTACAGCCTCAGCGAGCC	CATGCGCACCTCGTCCAG	1441–1717	276
UL97_8	GTAACGTGCTGGGCTTTTGC	GCATTCGTGGTAGAAGCGGC	1670–1949	279
UL97_9	CCAAGATGTCCTCGTGTCGC	CCAGGTTACTCGGGGAACAG	1916–2129	184

### Sanger DNA sequencing and data analysis

Sanger sequencing was performed using an Applied Biosystems 3130 and 3500xL Genetic Analyzer (ThemoFisher Scientific, Waltham, MA). Since we used formalin-fixed paraffin-embedded (FFPE) samples that are prone to false-positive mutation findings, we performed at least two independent DNA extractions and confirmed that the results were the same. In the DNA fragmentation check, we determined by electrophoresis to be suitable samples if all target PCR products were properly amplified. Both sense and antisense strands were analyzed independently to exclude false-positive mutations, and it was confirmed that both results were in agreement. Established sequences were compared to the UL97 gene of the wild-type GCV-sensitive AD169 strain (GenBank accession No. BK000394) as a reference.

### Statistical analysis

Statistical analysis was performed using statistical software (SPSS for Windows, version 16.0; SPSS Inc., Chicago, IL). The Fisher’s exact test was used to compare categorical variables between the UC and non-UC groups. The Mann–Whitney *U*-test and independent Student’s *t*-test were used to compare continuous variables of the UC and non-UC groups, as appropriate. The Fisher's exact test was used to compare each mutation frequency in the UL97 gene between the UC and non-UC groups. A *P* value of < 0.05 was considered significant.

## Results

### Patient characteristics

The demographic information for 22 GCV-naive patients enrolled in this study is shown in Table 2. The mean age was 59.5 years. Fifteen patients had UC and seven patients had other diseases. In non-UC patients, the background diseases were malignant melanoma (one patient), myelodysplastic syndrome (one patient), sarcoidosis (one patient), ischemic colitis (one patient), autoimmune hepatitis (one patient), and hemodialysis (one patient). A comparison of the patient characteristics between the UC and non-UC groups are shown in Table 3. In the comparison of the two groups, serum albumin was significantly lower in the non-UC group. Among the treatments, there was a significant difference only in the use of 5-aminosalicylate (5-ASA) (Table 3). Among the UC patients, steroid therapy was used for five patients (33.3%), immunosuppressive therapy was used for seven patients (46.7%), anti-TNF therapy was used for five patients (33.3%), and 5-ASA was used for 10 patients (66.7%). The clinical UC activity of the enrolled patients assessed by the Rachmilewitz index was 8.75 ± 5.39 (Supplementary Table S1).

**Table 2:** Enrolled patients and their primary diseases.

Number of patients, n	22
Sex, men/women	13/9
Age, y, mean ± SD (range)	59.5 ± 16.0 (17–81)
**Primary diseases, n (%)**	
Ulcerative colitis	15 (68.2)
Non-ulcerative colitis	7 (31.8)
Malignant melanoma	1 (4.5)
Myelodysplastic syndrome	1 (4.5)
Sarcoidosis	1 (4.5)
Ischemic colitis	1 (4.5)
Autoimmune hepatitis	1 (4.5)
Hemodialysis	1 (4.5)
No disease	1 (4.5)

**Table 3:** Comparison of the patient characteristics between the UC and non-UC groups.

	Overall	UC	Non-UC	Statistical analysis
Number of patients, n	22	15	7	
Sex, men/women	13/9	9/6	4/3	NS
Age; mean ± SD (range), years	59.5 ± 16.0 (17–81)	56.1 ± 17.8 (17–81)	66.9 ± 8.4 (53–76)	NS
Height, cm, mean ± SD	161.0 ± 8.7	160.9 ± 8.3	161.4 ± 10.0	NS
Weight, kg, mean ± SD	52.8 ± 8.6	52.7 ± 9.9	52.9 ± 5.5	NS
Body mass index, mean ± SD	20.4 ± 3.1	20.3 ± 3.1	20.5 ± 3.5	NS
Serum albumin, g/dl, mean ± SD	3.11 ± 0.77	3.42 ± 0.65	2.46 ± 0.59	*P* < 0.01
Serum CRP, mg/dl, mean ± SD	2.43 ± 2.21	2.15 ± 2.45	3.01 ± 1.59	NS
Hemoglobin, g/dl, mean ± SD	11.9 ± 2.4	12.3 ± 2.6	11.0 ± 1.7	NS
Total cholesterol, mg/dl, mean ± SD	163 ± 45	157 ± 36	171 ± 56	NS
Triglyceride, mg/dl, mean ± SD	121 ± 50	106 ± 39	140 ± 60	NS
**Treatment, n (%)**				
Oral or intravenous steroids	8 (36.4)	5 (33.3)	3 (42.9)	NS
Immunosuppressive agents	9 (40.9)	7 (46.7)	2 (28.6)	NS
Anti-TNF agents	5 (22.7)	5 (33.3)	0 (0)	NS
5-ASA	10 (45.5)	10 (66.7)	0 (0)	*P* < 0.01
Immune checkpoint inhibitor	1 (4.5)	0 (0)	1 (14.3)	NS
Other	8 (36.4)	5 (33.3)	3 (42.9)	NS
**Diagnosis of CMV infection, n (%)**				
Histological H&E staining	4 (18.2)	0 (0)	4 (57.1)	*P* < 0.01
Histological IHC staining	19 (86.4)	13 (86.7)	6 (85.7)	NS
Tissue CMV DNA PCR	22 (100)	15 (100)	7 (100)	NS
**Sample, n (%)**				
Frozen sample	7 (31.8)	5 (33.3)	2 (28.6)	NS
FFPE sample	15 (68.2)	10 (66.7)	5 (71.4)	

*H&E* hematoxylin and eosin, *IHC* immunohistochemistry, *FFPE* formalin-fixed paraffin-embedded.

### Diagnosis of CMV infection in the colon

In our study, CMV infection in the colon was diagnosed on the basis of mucosal biopsies with macroscopic inflammation. Infected cells with intracellular inclusion bodies were detected in 4 of 22 patients by hematoxylin and eosin staining in tissue specimens (Table 3). Histological IHC staining revealed that 19 of 22 patients were CMV positive. All patients were CMV positive as determined by the tissue CMV DNA PCR method.

### Prevalence of the UL97 gene mutation

**Figure 2:**
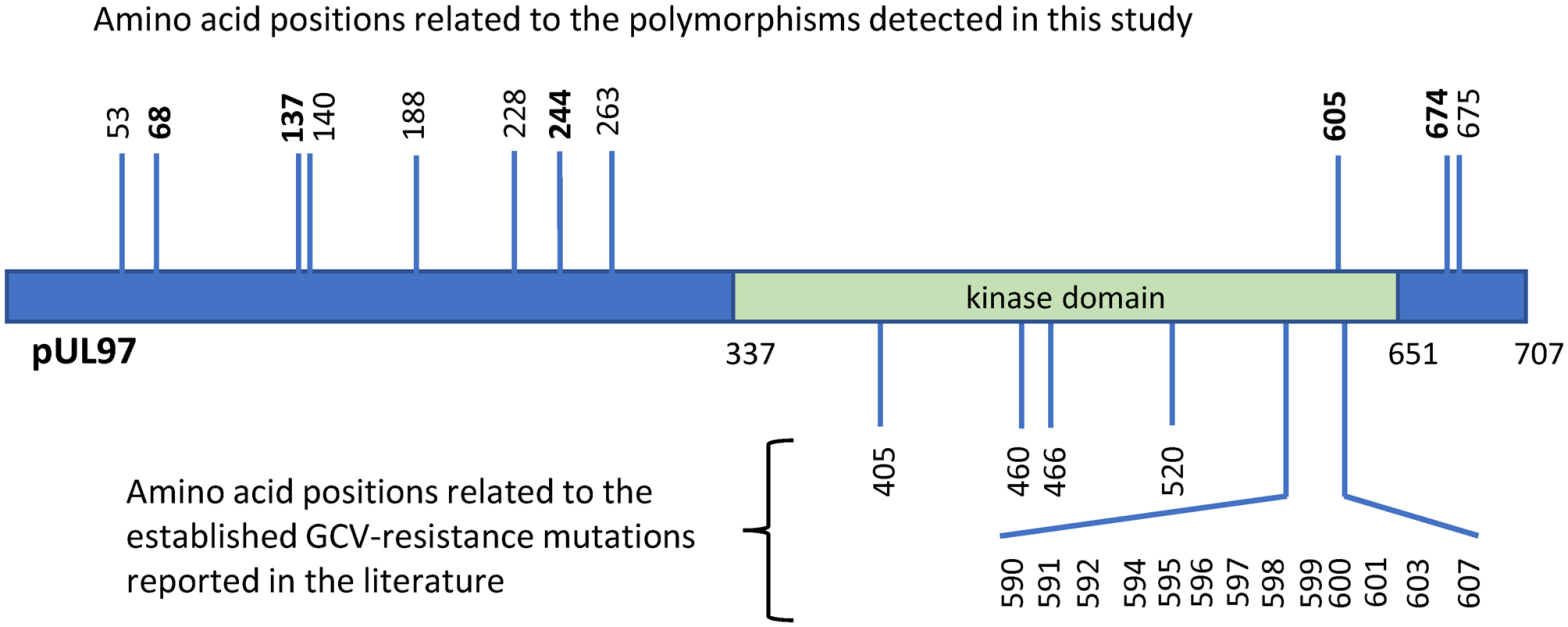
Schematic diagram of natural polymorphisms and GCV-resistant mutations in pUL97. The kinase domain is located between amino acids 337 and 651. All amino acid positions related to the established GCV-resistance mutations reported in the literature are indicated below. Interaction region for GCV is defined by the location of resistance mutations detected within the kinase domain so far (405, 460, 466, 520, 590, 591, 592, 594, 595, 596, 597, 598, 599, 600, 601, 603, 607)^34^. Amino acid positions related to polymorphisms detected in this study are indicated above. Natural polymorphisms that have been reported in the literature are indicated in bold^32^.

To identify the mutations of the UL97 gene in GCV-naive patients, Sanger DNA sequencing of the PCR products was performed. Compared with the wild-type GCV-sensitive AD169 strain as a reference, we were able to detect several polymorphisms of the UL97 gene. Overall, in 22 patients with CMV reactivation in the colon, natural polymorphisms were frequently detected in codons N68D (100%), I244V (100%), and D605E (86.4%) in comparison with AD169 reference strain. Seven polymorphisms were detected infrequently (< 10%) in codons A53S, R137C, A140V, G188S, L228P, D263G, A674T, and T675A, which were located outside the kinase domain (Table 4). However, no known GCV resistance mutations were found in our series (Fig. 2).

**Table 4:** Amino acid changes related to UL97 mutations and polymorphisms in 22 Japanese patients with colonic CMV infection in comparison with AD169 reference strain.

Amino acid changes	Overall		UC		Non-UC		Statistical analysis
	No. of strains	% of strains	No. of strains	% of strains	No. of strains	% of strains	
A53S	1	4.5	1	6.7	0	0	NS
N68D	22	100.0	15	100.0	7	100	NS
R137C	1	4.5	0	0	1	14.3	NS
A140V	1	4.5	1	6.7	0	0	NS
G188S	1	4.5	0	0	1	14.3	NS
L228P	2	9.0	1	6.7	1	14.3	NS
I244V	22	100	15	100	7	100	NS
D263G	2	9.0	1	6.7	1	14.3	NS
D605E	19	86.4	14	93.3	5	71.4	NS
A674T	1	4.5	0	0	1	14.3	NS
T675A	1	4.5	1	6.7	0	0	NS

### Comparison between UC and non-UC patients

We attempted to examine whether the frequency and type of UL97 polymorphisms in GCV-naive patients were different between UC and non-UC patients. As shown in Table 4, the ratio of the polymorphisms seemed to be similar between the UC group and non-UC group although the number of samples was insufficient for statistical analysis.

## Discussion

In the present study, we investigated the prevalence of CMV UL97 gene mutations and polymorphisms in Japanese patients with CMV infection in the colon for the first time. We revealed that several UL97 polymorphisms were detected, such as in codons N68D (100%), I244V (100%), and D605E (86.4%). However, no known GCV resistance mutations were found in our series of GCV-naive patients. Furthermore, there seemed to be no difference between the ratio of polymorphisms in the UC and the non-UC patients. Consistent with previous reports, the D605E polymorphism could be used as a genetic marker for CMV in East Asian countries.

Human CMV remains the most common infection in solid organ recipients, HCT recipients, HIV-infected patients, and children with congenital immunodeficiencies. It remains an important pathogen despite advances in the prophylaxis and acute treatment of CMV. The emergence of CMV resistance in a patient reduces the clinical efficacy of antiviral therapy, complicates therapeutic and clinical management decisions, and, in some cases, results in death of the patient. According to recent reports, the incidence of GCV resistance is 5–12% among solid organ recipients^21–23^ and 31% in intestinal and multivisceral organ transplant recipients^24^. GCV resistance is 7.9% in HCT recipients from matched related or unrelated donors^22,25^ and 14.5% in high-risk patients^26^. GCV resistance in HIV-infected patients is reported to be 19.5%^27^.

Common mechanisms of CMV resistance to GCV have been described chiefly with UL97 mutations. In several reports, numerous GCV-related mutations have been described. Most UL97 mutations conferring GCV resistance are strongly clustered at codons 460, 520, or 590 to 607^16,28,29^. In daily medical care, timely results of resistance testing would be useful for making clinical decisions. If no drug resistance is identified, clinical management may focus on improving host defenses rather than switching antivirals. If there is confirmed genotypic evidence of resistance, the specific mutation, host immune status, and disease severity should all factor into these decisions, to continue or intensify current treatment, to switch to a non-cross-resistant drug, to use drug combinations, or to try experimental drugs. Management algorithms have been proposed by several groups^30^.

In this study, we did not detect the mutations of the UL97 gene associated with GCV resistance. Our results might indicate that the uniform use of GCV is appropriate for initial antiviral therapy in CMV colitis associated with or without UC in GCV-naive patients, when antiviral therapy is needed. Since the sample size in our study was small, further study with large sample size is necessary to confirm it. On the other hand, several polymorphisms of the UL97 gene not associated with GCV resistance were detected. Frequently detected polymorphisms in codons N68D (100%), I244V (100%) have been confirmed in previous reports^31–33^. Seven polymorphisms were detected infrequently (< 10%). These polymorphisms were located outside the interaction region for GCV in the kinase domain^34^. The polymorphism of D605E (86.4%) was located within the kinase domain and reported as GCV-sensitive polymorphism^35–37^. The D605E variant of UL97 was first described in 1 of 8 CMV isolates from an immunocompromised host in France^38^, but it has not been commonly observed in the human CMV strains circulating in western countries. Some reports have shown that the D605E has frequently been detected in only Asian countries, and is estimated at 91.8% in Japanese infants and children and 78% in Chinese transplant recipients^17,18^. Thus, they suggested that this variant could be an important genetic marker of CMV evolution in East Asian countries. The high frequency of D603E in our study was consistent with their results.

There are several limitations in this preliminary study. First, this study included a small number of patients, all of whom were GCV-naive patients. Therefore, the results should be interpreted only in GCV-naive patients. It is speculated that a high rate of UL97 mutations may be identified in patients who have been treated with GCV for a longer period. Second, FFPE samples were used because there were not many cases with colonic CMV infection in daily clinical practice. False-mutation identification has been avoided with possible methods and it was confirmed that there was no difference in the frequency of polymorphisms between FFPE and frozen tissue sample (Supplementary Table S2). However, it might not be completely ruled out. Third, we used the Sanger sequence to detect the mutations in this study. This cannot detect mutants that are present in < 10%–20% of the viral population. Recently, several studies have been reported which detected the mutation of the CMV gene using a next-generation sequence (NGS)^39,40^. NGS methods have an improved ability to detect mixed populations and have been used to assess low-abundance variants. Forth, we did not assess the mutation of DNA polymerase UL54 gene in this study, which is related to GCV resistance and cross-resistance to other antiviral drugs^41^. Therefore, further studies using NGS targeting both UL97 and UL54 in patients who have been treated with GCV are expected to provide more reliable evidence than this study.

Recognizing these limitations, the implications of our report are that no drug-resistant CMV strains were detected in the mucosa of the colon in patients with gastrointestinal CMV infection without a history of GCV administration, suggesting that it might not be necessary to consider CMV drug resistance at treatment initiation. In addition, these results were similarly observed when associated with UC and non-UC disease. Furthermore, D605E polymorphism could potentially be used as a genetic marker for CMV in East Asian countries. Since GCV-resistant CMV infection is a problem associated with poor prognosis in fulminant UC patients in clinical practice, it would be important to investigate the frequency of drug resistance mutations after GCV administration in patients with these conditions as the next step. We hope that our study will lead to further clinical research in this area.

## Supplementary information

Supplementary Information.

## Abbreviations

CMV: Cytomegalovirus
UC: Ulcerative colitis
GCV: Ganciclovir
HIV: Human immunodeficiency virus
PCR: Polymerase chain reaction
FFPE: Formalin-fixed paraffin-embedded
DNA: Deoxyribonucleic acid
NGS: Next-generation sequencing
