# Supplementary material for: Prevalence of UL97 gene mutations and polymorphisms in cytomegalovirus infection in the colon associated with or without ulcerative colitis
Source: Sci Rep. 2021 Jul 1;11:13676. doi: 10.1038/s41598-021-93168-x (PMC8249415; doi:10.1038/s41598-021-93168-x)
Supplement: Supplementary file 1 — Supplementary Information. [file 41598_2021_93168_MOESM1_ESM.docx]

**Prevalence of UL97 gene mutations and polymorphisms in cytomegalovirus infection in the colon associated with or without ulcerative colitis**

Satoshi Tamura^1, 2^, Satoshi Osawa^1^, Natsuki Ishida^2^, Takahiro Miyazu^2^, Shinya Tani^2^, Mihoko Yamade^2^, Moriya Iwaizumi^3^, Yasushi Hamaya^2^, Isao Kosugi^4,^ Takahisa Furuta^5^, and Ken Sugimoto^2^

^1^ Department of Endoscopic and Photodynamic Medicine, Hamamatsu University School of Medicine, 1-20-1 Handayama, Higashi-ku, Hamamatsu 431-3192, Japan

^2^ First Department of Medicine, Hamamatsu University School of Medicine, 1-20-1 Handayama, Higashi-ku, Hamamatsu 431-3192, Japan

^3^ Department of Laboratory Medicine, Hamamatsu University School of Medicine, 1-20-1 Handayama, Higashi-ku, Hamamatsu 431-3192, Japan

^4^ Department of Regenerative and Infectious Pathology, Hamamatsu University School of Medicine, 1-20-1 Handayama, Higashi-ku, Hamamatsu 431-3192, Japan

^5^ Center for Clinical Research, Hamamatsu University School of Medicine, 1-20-1 Handayama, Higashi-ku, Hamamatsu 431-3192, Japan

**Supplementary Information**

**Supplementary Table S1** Patient characteristics with ulcerative colitis

| Number of Patients | 15 |
| --- | --- |
| Gender, male/female | 9/6 |
| Age, y, mean ± SD (range) | 48.6 ± 13.3 (24-67) |
| Disease duration, y, mean ± SD (range) | 13.9 ± 9.5 (1-32) |
| Type of disease, n (%)  　Total colitis  　Left-sided  　Proctitis | 9 (60)  3 (20)  3 (20) |
| Inpatient/Outpatient | 9/6 |
| Disease activity, Rachmilewitz index, mean ± SD (range) | 8.75 ± 5.39 (0-16) |
| Serum albumin, g/dl, mean ± SD | 3.42 ± 0.65 |
| Serum CRP, mg/dl, mean ± SD | 0.82 ± 0.97 |
| Treatment, n (%)  Oral or intravenous steroids  Immunosuppressive agents  Anti-TNF agents  5-ASA  Other  No treatment | 5 (33.3)  7 (46.7)  5 (33.3)  10 (66.7)  5 (33.3)  1 (6.7) |
| Outcome  　Recovery  　Colectomy | 13 (86.7)  2 (13.3) |

**Supplementary Table S2** UL97 polymorphisms in 22 Japanese patients with colonic CMV infection in comparison with AD169 strain; FFPE sample vs frozen biopsy sample

| Amino acid changes | Overall | | UC | | Non-UC | | Total number of amino acid changes per total codons | Frequency of amino acid changes per total codons (%) |
| --- | --- | --- | --- | --- | --- | --- | --- | --- |
|  | No. of strains | % of strains | No. of strains | % of strains | No. of strains | % of strains |  |  |
| **FFPE sample** | **15** |  | **10** |  | **5** |  | 50 / 10,605 | **0.471** |
| A53S | 1 | 6.7 | 1 | 10 | 0 | 0 |  |  |
| N68D | 15 | 100 | 10 | 100 | 5 | 100 |  |  |
| R137C | 1 | 6.7 | 0 | 0 | 1 | 20 |  |  |
| A140V | 1 | 6.7 | 1 | 10 | 0 | 0 |  |  |
| V244I | 15 | 100 | 10 | 100 | 5 | 100 |  |  |
| L228P | 2 | 13.3 | 1 | 10 | 1 | 14.3 |  |  |
| D263G | 2 | 13.3 | 1 | 10 | 1 | 14.3 |  |  |
| D605E | 12 | 80 | 9 | 90 | 3 | 60 |  |  |
| T675A | 1 | 6.7 | 1 | 10 | 0 | 0 |  |  |
| **Frozen biopsy sample** | **7** |  | **5** |  | **2** |  | 23 / 4,949 | **0.465** |
| N68D | 7 | 100 | 5 | 100 | 2 | 100 |  |  |
| G188S | 1 | 14.3 | 0 | 0 | 1 | 50 |  |  |
| V244I | 7 | 100 | 5 | 100 | 2 | 100 |  |  |
| D605E | 7 | 100 | 5 | 100 | 2 | 100 |  |  |
| A674T | 1 | 14.3 | 0 | 0 | 1 | 50 |  |  |

FFPE; formalin-fixed paraffin-embedded
